# Interaction of *APOE4* alleles and PET tau imaging in former contact sport athletes

**DOI:** 10.1016/j.nicl.2020.102212

**Published:** 2020-02-13

**Authors:** Anna Vasilevskaya, Foad Taghdiri, Charles Burke, Apameh Tarazi, Seyed Ali Naeimi, Mozghan Khodadadi, Ruma Goswami, Christine Sato, Mark Grinberg, Danielle Moreno, Richard Wennberg, David Mikulis, Robin Green, Brenda Colella, Karen D. Davis, Pablo Rusjan, Sylvain Houle, Charles Tator, Ekaterina Rogaeva, Maria C. Tartaglia

**Affiliations:** a Tanz Centre for Research in Neurodegenerative Diseases, University of Toronto, 60 Leonard avenue, Toronto, ON M5T 0S8, Canada; b Institute of Medical Science, University of Toronto, 1 King's College Circle, Toronto, ON, M5S 1A8, Canada; c Division of Neurology, Toronto Western Hospital, University Health Network, 399 Bathurst St., Toronto, ON, M5T 2S8, Canada; d Canadian Concussion Center, Toronto Western Hospital, Krembil Neuroscience Centre, University Health Network, 399 Bathurst St., Toronto, ON, M5T 2S8, Canada; e School of Medicine & Dentistry, Western University, Windsor, ON, Canada; f Division of Neuroradiology, Joint Department of Medical Imaging, University Health Network, 399 Bathurst St., Toronto, ON, M5T 2S8, Canada; g Department of Rehabilitation Sciences, University of Toronto, 500 University Ave, Toronto, ON, M5G 1V7, Canada; h Department of Surgery, University of Toronto, 149 College St., Toronto, ON, M5T 1P5, Canada; i Research Imaging Centre, Campbell Research Institute, Centre for Addiction and Mental Health, 250 College St., Toronto, ON, M5T 1R8, Canada; j Division of Neurosurgery, Toronto Western Hospital, Krembil Brain Institute, University Health Network, 399 Bathurst St., Toronto, ON, M5T 2S8, Canada; k Department of Medicine, Division of Neurology, University of Toronto, 1 King's College Circle, Toronto, ON, M5S 1A8, Canada

**Keywords:** Concussion, Apoe4, Positron emission tomography, Tau, Chronic traumatic encephalopathy

## Abstract

**Background:**

Genetic polymorphisms like apolipoprotein E (*APOE*) and microtubule-associated protein tau (*MAPT*) genes increase the risk of neurodegeneration.

**Methods:**

38 former players (age 52.63±14.02) of contact sports underwent neuroimaging, biofluid collection, and comprehensive neuropsychological assessment. The [F-18]AV-1451 tracer signal was compared in the cortical grey matter between *APOE4* allele carriers and non-carriers as well as carriers of *MAPT* H1H1 vs non-H1H1. Participants were then divided into the high (*N* = 13) and low (*N* = 13) groups based on cortical PET tau standard uptake value ratios (SUVRs) for comparison.

**Findings:**

Cortical grey matter PET tau SUVR values were significantly higher in *APOE4* carriers compared to non-carriers (*p* = 0.020). In contrast, there was no significant difference in SUVR between *MAPT* H1H1 vs non-H1H1 carrier genes (*p* = 1.00). There was a significantly higher *APOE4* allele frequency in the high cortical grey matter PET tau group, comparing to low cortical grey matter PET tau group (*p* = 0.048). No significant difference in neuropsychological function was found between *APOE4* allele carriers and non-carriers.

**Interpretation:**

There is an association between higher cortical grey matter tau burden as seen with [F-18]AV-1451 PET tracer SUVR, and the *APOE4* allele in former professional and semi-professional players at high risk of concussions. *APOE4* allele may be a risk factor for tau accumulation in former contact sports athletes at high risk of neurodegeneration.

**Funding:**

Toronto General and Western Hospital Foundations; Weston Brain Institute; Canadian Consortium on Neurodegeneration in ageing; Krembil Research Institute. There was no role of the funders in this study.

## Highlights

• Cortical PET tau was compared between APOE4 carriers and non-carriers.
• APOE4 carriers had higher cortical PET tau comparing to non-carriers.
• APOE4 as a risk factor for tau accumulation in former contact sports athletes.

## 1 Introduction

Chronic traumatic encephalopathy (CTE) is a progressive neurodegenerative disease believed to be associated with repetitive head impacts. Retrospective analysis of pathological case series revealed that CTE was associated with irritability, impulsivity, aggression, depression, memory and cognitive impairments, as well as heightened suicidality [McKee et al., 1, McKee et al., 2009, Omalu et al., Jun, Hazrati et al., 2013, Omalu et al., 1]. Although the pathological changes of CTE were originally described in boxers [Martland, 1928 Oct 13, Critchley, 1949, Millspaugh, 1937], confirmed CTE cases come from a variety of contact sports including American football, hockey, wrestling, and soccer; as well as from military personnel and non-sport related concussions [McKee et al., 2009, Tartaglia et al., 2014, Smith et al., Apr]. Two recent studies found strong dose-response relationships between number of years played contact sports and CTE neuropathology [Mez et al., 2020, Stern et al., 2019]. The clinical and pathological presentations of CTE overlap with those of Alzheimer's disease (AD) and frontotemporal lobar degeneration, but CTE pathology has its own distinct features [McKee et al., 2009, Tartaglia et al., 2014]. The pathognomonic lesion of CTE, as defined by a National Institute of Neurological Disease and Stroke (NINDS)/National Institute of Biomedical Imaging and Bioengineering (NIBIB) meeting, consists of irregular hyperphosphorylated tau deposits in neurons and astroglia, preferentially at the depths of the sulci in the superficial cortical layers and around blood vessels [McKee et al., 1]. β-amyloid and TAR DNA-binding protein 43 (TDP-43) inclusions are also reported in some studies [McKee et al., 1, McKee et al., 2009, Omalu et al., Jun, Hazrati et al., 2013, Omalu et al., 1, Gavett et al., 1, Corsellis et al., Aug, 16, Corsellis and Brierley, 1959 Jul, Geddes et al., 1, Stern et al., 1, Saing et al., 21, Hof et al., 1, Mez et al., 25, Bieniek et al., 1, Goldstein et al., 16, 25]. There are currently no ante-mortem biomarkers for the tau pathology of CTE, and the diagnosis is made based on post-mortem neuropathological examination of the brain tissue.

Phosphorylated tau, the pathological substrate of CTE is similar to that observed in Alzheimer's disease but has its own distinct features [Falcon et al., Apr]. The use of positron emission tomography (PET) imaging with [F-18]AV-1451 ([F-18]T807; Flortaucipir, AVID Radiopharmaceuticals), a tau specific tracer, allows the detection of abnormal aggregates of phosphorylated tau protein in vivo in AD [Marquié et al., 2015]*.* Its use in AD has been widely examined and tracer retention correlated with post mortem neurofibrillary tangles (NFTs) containing tau in the form of paired helical filaments [Marquié et al., 2015, Lemoine et al., 2017, Jovalekic et al., 2017, Xia et al., 1]. As well, binding was higher in AD patients than in patients with mild cognitive impairment or healthy controls, and tracer binding was associated with worsening cognitive function [Cho et al., 2016]. PET imaging with [F-18]AV-1451 tau specific tracer shows promise as a potential in vivo biomarker of CTE pathology, however its ability to reliably detect CTE lesions is unclear and requires more investigation [Robinson et al., 3, Lesman-Segev et al., 1, Marquié et al., 28]. One study reported mildly elevated PET tau binding in two out of nine amyloid negative patients at risk for CTE, with the distribution pattern consistent with CTE pathology stages III-IV. This result suggests PET tau might not be sensitive to CTE lesions in early disease stages [Lesman-Segev et al., 1]. Earlier case reports of this tracer in formerly concussed athletes presented cases of former National Football League (NFL) players with a history of multiple concussions [Mitsis et al., 2014, Dickstein et al., Sep]. The first case was of a 71-year-old with memory impairments and a clinical profile similar to AD. The amyloid PET scan was negative so no evidence of AD pathology. The PET tau tracer [F-18]AV-1451 showed predominantly subcortical signal, with the highest signal coming from the basal ganglia and substantia nigra [Mitsis et al., 2014]. Tracer retention in the basal ganglia and substantia nigra regions has previously been pathologically confirmed to be off-target binding [Marquié et al., 2015], but a more recent study described the basal ganglia binding to be correlated with age-related iron accumulation in that region [Marquié et al., 2015, Choi et al., 1]. The second case of [F-18]AV-1451 tracer binding was in a 39 year old athlete with progressive neuropsychiatric issues – specifically emotional lability and irritability. The amyloid scan was negative, largely ruling out AD pathology, and the PET [F-18]AV-1451 tau scan showed a higher tracer signal in the cortex [Dickstein et al., Sep]. Other signal increases were noted in the midbrain, globus pallidus, and the hippocampus, with the midbrain and globus pallidus being pathologically confirmed off-target binding sites [Marquié et al., 2015, Dickstein et al., Sep, Choi et al., 1]. Another study examined the use of the same PET tau tracer in veterans with blast neurotrauma, and found increased tracer signal in the frontal, occipital, and cerebellar brain regions [Robinson et al., 3]. Finally, a more recent cohort study using [F-18]AV-1451 PET tau tracer found increased bilateral superior frontal, bilateral medial temporal and left parietal SUVRs in 26 former National Football League players comparing to 31 controls. Tau SUVRs in these regions correlated with total years of tackle football amongst the former players cohort [Stern et al., 2019].

Even though the exact CTE incidence amongst athletes is unclear, not all individuals with exposure to contact sports and repetitive head impacts develop CTE [Hazrati et al., 2013, Omalu et al., 1, Mez et al., 25, Bieniek et al., 1]. Genetics might play a role in increasing CTE susceptibility. There is growing evidence that some genetic polymorphisms increase the risk of neurodegenerative diseases [Zhang et al., 29, Corder et al., 1993]. Allelic variants of the apolipoprotein E (*APOE*) gene have been implicated in a number of neurodegenerative diseases [Giau et al., 16]. The two missense polymorphisms in *APOE* underly the three molecular isoforms [Mahley and Apolipoprotein, 2016 Jul 1]: *APOE* epsilon 2 (ε2), *APOE* epsilon 3 (ε3), and *APOE* epsilon 4 (ε4). *APOE4* has been shown to increase the risk of AD [Corder et al., 1993, Mahley and Apolipoprotein, 2016 Jul 1]. The exact mechanism by which *APOE4* influences AD risk is not yet understood, however increasing evidence points to the amyloid hypothesis – where *APOE4* directly, and indirectly influences amyloid beta metabolism [Kanekiyo et al., 2014]. The relationship between *APOE* alleles and tau pathology is less clear. Some authors propose an interaction between amyloid and tau proteins in the brain, where amyloid fibrils increase tau phosphorylation and aggregation [Adalbert et al., 2007]. Therefore, *APOE4* may have an indirect effect on tau accumulation through amyloid. However, some in vitro animal studies demonstrated a direct effect of *APOE* on tau pathogenesis [Strittmatter et al., 1994, Shi et al., 2017]. In context of traumatic brain injuries (TBIs), *APOE4* is associated with poor clinical outcomes in patients with TBIs [Mahley and Apolipoprotein, 2016 Jul 1]. Additionally, the *APOE4* allele has been associated with elevated post-concussion symptoms in military veterans [Merritt et al., 21], and increased phosphorylated tau levels in the brains of a blast-injury mouse model [Cao et al., 12]. This provides limited, but possible evidence for an association between *APOE* and tau pathology in TBI cases.

Another polymorphism implicated in neurodegeneration is in the microtubule-associated protein tau (*MAPT*) gene, which is responsible for the production of tau protein [Pittman et al., 15]. Mutations in the *MAPT* gene may lead to abnormal structure and function of tau, and currently almost 60 *MAPT* mutations are linked to neurodegeneration. There are two main *MAPT* haplotypes – H1 and H2 [Zhang et al., 29, Zhang et al., 1]. The H1 haplotype is associated with an increased risk of developing 4-repeat tauopathies – progressive supranuclear palsy (PSP) and corticobasal degeneration (CBD). Previous research highlighted that the H1 haplotype is significantly overrepresented in pathologically confirmed CBD and PSP populations, compared to controls [Houlden et al., 2001, Pastor et al., Aug, Pittman et al., 15, Baker et al., 1]. The literature examining *MAPT* haplotypes in relation to head impacts and CTE is limited, however one study found a slight increase in frequency of *MAPT* H1/H1 genotype in men with contact sports exposure and confirmed CTE pathology, comparing to men with contact sports exposure without CTE pathology and to clinical controls [Bieniek et al., 1].

This study examines the effect of the *APOE4* allele and *MAPT* H1H1 on SUVRs of PET tau-specific [F-18]AV-1451 tracer in former professional contact sport athletes at risk for CTE. We hypothesize that carriers of *APOE4* allele and/or H1H1 carriers will have higher PET [F-18]AV-1451 signal.

## 2 Methods

### 2.1 Participants

Thirty-eight athletes engaged in sports with high risk of concussions were included as part of this ongoing study. The recruitment was completed through the Canadian Football League (CFL) Alumni Association and the Toronto Western Hospital (Toronto, Canada) concussion clinic. Inclusion criteria are participants under 85 years old who are fluent in English and are former professional or semi-professional sport athletes at high risk of concussions. Exclusion criteria included the diagnosis of a neurological or psychotic disorder prior to the concussions, systemic illnesses affecting the brain, or lesions seen on magnetic resonance imaging (MRI). Due to the invasiveness of the procedure, only nine of 38 participants agreed to undergo a lumbar puncture so their CSF could be tested for AD biomarkers. For participants with no CSF available – structural MRI scans and PET tau imaging were examined by a cognitive neurologist (MCT) for evidence of AD pattern. All participants underwent comprehensive neuropsychological and neurological assessments, neuroimaging and blood collection during the same consecutive two-day visit. The study was approved by the Research Ethics Board of the University Health Network and written consent was obtained from all participants. Concussion exposure was determined based on the player's recall of injury using the concussion definition provided by the Concussion in Sport Group, as detailed in their most recent consensus statement on concussion in sport [McCrory et al., 2016]. In addition, all players underwent a semi-structured interview to verify the information and to jog memory for any events they may not have recalled.

### 2.2 Biofluid collection and genetics

Lumbar puncture for CSF collection was performed following AD Neuroimaging Initiative (ADNI) protocol [Jack Jr et al., 2008]. After CSF collection into polypropylene tubes, a sandwich ELISA method was used to measure Aβ_42_, phosphorylated tau (p-tau) and total tau (t-tau) levels according to the manufacturer's instructions [Maddalena et al., 2003]. AD pathology was considered present if p-tau > 68 pg/ml and Aβ_42_ to t-tau index < 0.8 [Blennow et al., 2015].

Blood was collected from all participants and genomic DNA was extracted using a Qiagen kit from whole blood. The *APOE* genotypes and *MAPT* haplotypes were determined as previously described [Saunders et al., 1993].

### 2.3 Neuroimaging

PET tau imaging with 5mCi of [F-18]AV-1451 tracer was performed. Thirty-six participants were scanned using a Biograph HiRez XVI PET/CT scanner (Siemens Molecular Imaging, Knoxville, TN, USA), while 2 participants were scanned using a 3D High Resolution Research Tomograph (HRRT) (CPS/Siemens, Knoxville, TN, USA) PET scanner. Following a 45-minute uptake time, static PET images (45–120 min) were acquired for a duration of 75 min. T1 structural MRI images were acquired using a 3T GE Signa scanner with 8 channel headcoil and the following scan parameters: TE=5 ms, TR=12 ms, flip angle = 45°; 128 axial slices, slice thickness=1.5 mm, 256 × 256 matrix, FOV=24 × 24 cm. The region of interest (ROI) analysis was completed on the PET data using in-house ROMI software using the ROI delineation method as previously described [Rusjan et al., 30]. The PET images were corrected for head motion and partial volume effect [Müller-Gärtner et al., 1992]. For a single ROI of the cortical grey matter (excluding cerebellum), SUVRs were calculated from the PET data between 50 and 80 min and, in a subset of the participants, from the data between 80–100 min post injection. The cerebellar grey matter was used as the reference region.

### 2.4 Neuropsychological testing

The following tests, with known sensitivity to TBIs and neurodegeneration were used for this study: trail making test (TMT) parts A and B [Kortte et al., 2002, Strauss et al., 2006], Rey auditory verbal learning test (RAVLT) [Schmidt, 1996] and Rey visual design learning test (RVDLT) [Strauss et al., 2006], symbol digit modalities test (SDMT) [Smith, 1982, Lezak et al., 2004] and digit span backward and forward [D. Wechsler, 1997]. Personality was assessed using the personality assessment inventory (PAI) [Morey, 1991]. The scores were standardized based on posted norms [Strauss et al., 2006, Smith, 1982, D. Wechsler, 1997, Geffen et al., 1990, Heaton, 1992]. The higher scores on TMT A & B, RAVLT, RVDLT, SDMT, digit span forward & backward assessments indicate better cognitive functioning, while higher scores on PAI depression and aggression assessments indicate higher levels of impairment. The cut-off threshold of 1.5 standard deviations below the mean was used to signify impaired functioning on TMT A & B, RAVLT, RVDLT, SDMT, digit span forward & backward assessments. The cut-off threshold of 1.5 standard deviations above the mean was used to signify impaired functioning on PAI aggression and depression assessments.

### 2.5 Statistical analysis

Statistical analysis was completed using IBM SPSS Statistics version 24 (IBM Corp., Armonk, NY, USA). All between-group demographics and neuropsychological testing comparisons were completed using an independent samples *t*-test, with the type of scanner comparison completed using Fisher's exact test. The number of concussions was not found to be normally distributed, therefore all between-group concussion number comparisons were completed using the Mann-Whitney U test. Due to the small sample size, participants had to be grouped based on *APOE4* carrier and non-carrier status. Regarding the *MAPT* gene – carriers of H1H2 and H2H2 diplotypes had to be grouped together and compared to carriers of H1H1 diplotype. The difference in mean cortical grey matter PET [F-18]AV-1451 SUVRs between carriers and non-carriers of specific alleles and diplotypes was determined using one-way ANCOVA, controlled for age. Neuropsychological assessment scores between *APOE4* carriers and non-carriers were also determined using an independent samples *t*-test. The cortical PET tau SUVR values amongst the study population presented on a continuum, ranging from 0.95 to 1.57. In order to compare the frequency of high risk allele *APOE4* in the lowest and the highest group based on PET tau SUVR values in the cortex, participants were divided into tertiles based on mean cortical PET [F-18]AV-1451 SUVR values, and the middle group was dropped from the analysis – leaving the high and low groups to be compared. We then completed a hypothesis driven comparison using Fisher's exact test of *APOE4* frequency between high and low cortical PET tau group, expecting a higher frequency of *APOE4* carriers amongst the high cortical grey matter PET tau group. Bonferroni correction was used to account for comparisons in mean cortical grey matter SUVR values between genotypes, and both adjusted and non-adjusted p-values are reported with a significance level set at *p*<0.05. For neuropsychological assessment score comparisons between genotypes, Bonferroni adjusted p-values with a significance level set at *p*<0.05 were reported only if any unadjusted p-values were significant at *p*<0.05.

## 3 Results

### 3.1 Participant demographics

Thirty-eight former athletes were included in this study (age 52.63±14.02; 37 males & 1 female). Amongst the cohort are 35 former professional athletes (2 National Hockey League & 29 CFL players, 3 boxers & 1 snow boarder), 3 semi-professionals (1 soccer, 1 hockey player, and 1 boxer). The number of professional years played by the professional athletes (*N* = 35) ranged from 2 to 21 (8.23±4.14). The semi-professional and amateur athletes (*N* = 3) had total years of play in contact sport ranging from 5 to 24 (14.33±9.50). The number of self-reported concussions for the whole cohort (*N* = 38) ranged from 0 to 60 (6.16±9.61). For those who had self-reported concussions (data presented for *N* = 35 because 2 participants did not recall any concussions and 1 participant did not remember the date of last concussion), the number of years since last reported concussion ranged from 0.5 to 61 years (20.90±16.27). The 2 participants with no reported concussions were included in the study because each had ≥10 years of play in contact sports and were very likely exposed to sub-concussive blows. Nine out of 38 participants who had cerebrospinal fluid (CSF) were AD negative. The remaining 29 participants were examined for the presence of AD-like pattern on MRI i.e. medial temporal atrophy and/or precuneus/posterior cingulate atrophy and on PET [F-18]AV-1451 SUVR for increased tracer uptake specifically in middle temporal lobe and posterior cortical regions including parietal lobe, and no such pattern was seen. Although cannot be ruled out entirely, AD pathology is unlikely in this cohort. The *APOE* genotype distribution of the entire cohort was as follows: 2 individuals with *APOE2*/*APOE4*, 5 individuals with *APOE3*/*APOE2*, 20 individuals homozygous for *APOE3*, 10 individuals with *APOE3*/*APOE4*, and 1 individual homozygous for *APOE4* allele. The *MAPT* diplotype distribution of the entire cohort was as follows: 21 individuals with H1H1, 14 individuals with H1H2 and 3 individuals with H2H2 diplotype.

### 3.2 Neuropsychological assessment results of the participant cohort

Overall, the participant cohort of this study showed to be quite high functioning with only a few individuals with impaired scores on neuropsychological testing. The distribution of performance on neuropsychological assessments was as follows: 1/38 participants had impaired performance on TMT A & B assessments; 1/37 participants had impaired performance on RAVLT, SDMT oral score, and digit span forward assessments; 7/37 participants had impaired performance on RVDLT assessment; 2/38 participants had impaired performance on SDMT written score; 5/38 participants had impaired performance on PAI depression and aggression assessments. Finally, no participants had impaired performance on digit span backward assessment. The impaired scores for each neuropsychological assessment between *APOE* and *MAPT* genotype groups are presented in Table 1 and 2. The impaired scores for each neuropsychological assessment for groups divided into tertiles based on cortical grey matter PET tau SUVR values are presented in Table 4 and 5.

**Table 1:** Demographics and neuropsychological assessments of *APOE4* allele carriers and non-carriers (mean ± standard deviation).

	***APOE4 Carrier***	***APOE4 Non-carrier***	***Unadjusted p***
***Demographics***			
N	13	25	
Age (years)	55.08±13.39	51.36±14.44	0.45
Education (years)	15.92±1.61	15.36±1.73	0.34
Concussion number	8.54±15.77	4.92±3.66	0.98
	(min:0, max:60,	(min:0, max:15,	
	median:4)	median:4)	
No. of professional years played (years)	6.77±4.62	8.00±4.56	0.44
	(min:0, max:15)	(min:0, max:21)	
No. of years since last reported concussion (years)	19.58±21.59^a^	21.59±15.82^b^	0.74
	(min:2, max:61)	(min:0.5, max:54)	
PET Scanner	12 PET/CT; 1 HRRT	23 PET/CT; 2 HRRT	1.00
***Neuropsychological Assessments***			
TMT A t-score	51.46±13.15	55.88±9.17	0.23
	(1/13 impaired)	(0/25 impaired)	
TMT B t-score	55.15±9.63	54.56±11.15	0.87
	(0/13 impaired)	(1/25 impaired)	
RAVLT Trials 1–5	0.29±0.85	−0.02±0.99^c^	0.35
z-score	(0/13 impaired)	(1/24 impaired)	
RVDLT Trials 1–5	0.36±1.64	−0.24±1.39^c^	0.25
z-score	(2/13 impaired)	(5/24 impaired)	
SDMT Oral z-score	0.45±1.48	0.67±1.51^c^	0.68
	(1/13 impaired)	(0/24 impaired)	
SDMT Written	0.29±1.40	0.30±0.74	0.98
z-score	(2/13 impaired)	(0/25 impaired)	
Digit Span	79.92±21.66	62.46±28.09^c^	0.06
Backwards%	(0/13 impaired)	(0/24 impaired)	
Digit Span Forward%	70.92±28.73	59.42±30.19^c^	0.27
	(0/13 impaired)	(1/24 impaired)	
PAI Depression	51.15±10.51	50.32±14.80	0.86
t-score	(2/13 impaired)	(3/25 impaired)	
PAI Aggression	50.92±11.16	52.44±11.23	0.69
t-score	(2/13 impaired)	(3/25 impaired)	

Independent student *t*-test, Fisher's exact test & Mann-Whitney U comparison; unadjusted significance level set at *p*<0.05 (2-sided). The number of participants with impaired scores is presented underneath the mean scores for each neuropsychological assessment in each group.

a Data is not included for 1 participant because he did not recall any concussions.

b Data is not included for 2 participants because 1 did not recall any concussions and 1 could not recollect the date of last concussion.

c One participant's score is missing due to the refusal to undergo the full neuropsychological testing, and a reduced battery was administered instead.

**Table 2:** Demographics of the *MAPT* H1H1 diplotype vs. H1H2/H2H2 diplotype (mean ± standard deviation).

	***H1H1 Diplotype***	***H1H2/H2H2 Diplotype***	***Unadjusted p***
***Demographics***			
N	21	17	
Age (years)	49.90±15.49	56.00±11.54	0.19
Concussion number	7.52±12.50	4.47±3.56	0.31
	(min:0, max:60,	(min:0, max:12,	
	median:4)	median:4)	
No. of professional years played (years)	8.05±5.28	7.00±3.55	0.49
	(min:0, max:21)	(min:0, max:14)	
No. of years since last reported concussion (years)	18.68±16.98^b^	23.87±15.34^a^	0.36
	(min:0.5, max:61)	(min:2, max:42)	
PET Scanner	18 PET/CT; 3 HRRT	17 PET/CT	0.24
***Neuropsychological Assessments***			
TMT A t-score	54.67±13.10	54.00±7.18	0.85
	(1/21 impaired)	(0/17 impaired)	
TMT B t-score	55.57±12.08	53.76±8.47	0.61
	(0/21 impaired)	(1/17 impaired)	
RAVLT Trials 1–5	−0.11±1.06	0.34±0.71^c^	0.15
z-score	(1/21 impaired)	(0/16 impaired)	
RVDLT Trials 1–5	0.00±1.40	−0.07±1.64^c^	0.89
z-score	(3/21 impaired)	(4/16 impaired)	
SDMT Oral z-score	0.43±1.22	0.81±1.79^c^	0.45
	(1/21 impaired)	(0/16 impaired)	
SDMT Written	0.42±0.90	0.15±1.11	0.41
z-score	(1/21 impaired)	(1/17 impaired)	
Digit Span	65.29±27.77	72.94±26.32^c^	0.40
Backwards%	(0/21 impaired)	(0/16 impaired)	
Digit Span Forward%	59.71±31.85	68.38±27.10^c^	0.39
	(1/21 impaired)	(0/16 impaired)	
PAI Depression	50.52±12.60	50.71±14.61	0.97
t-score	(3/21 impaired)	(2/17 impaired)	
PAI Aggression	53.71±12.29	49.71±9.25	0.27
t-score	(3/21 impaired)	(2/17 impaired)	

Independent student *t*-test, Fisher's exact test & Mann-Whitney U comparison; unadjusted significance level set at *p*<0.05 (2-sided). The number of participants with impaired scores is presented underneath the mean scores for each neuropsychological assessment in each group.

a Data is not included for 2 participants because 1 could not recall any concussions and 1 could not recollect the date of last concussion.

b Data is not included for 1 participant because he had no reported concussions.

c One participant's score is missing due to the refusal to undergo the full neuropsychological testing, and a reduced battery was administered instead.

### 3.3 Comparison between 50–80 and 80–100 min post-tracer injection time

All PET SUVR values reported were computed using 50–80 min post-tracer injection time. A subset of the participants (*N* = 24) had results available for 80–100 min post-tracer injection time, allowing for direct comparison between the time intervals. The cortical grey matter PET SUVRs were not found to be significantly different between the 2 time intervals for these 24 participants (*p*>0.4).

### 3.4 The relationship between *APOE4* and cortical grey matter PET tau

The *APOE4* carrier and non-carrier groups did not differ in demographics (Table 1). No difference in demographics was found between the *MAPT* H1H1 and H1H2/H2H2 diplotype groups (Table 2). One-way ANCOVA controlled for age showed a significant difference in cortical grey matter PET [F-18]AV-1451 SUVR values between the *APOE4* carrier and non-carrier groups (*p* = 0.010), however, no significant difference in SUVR were found in *MAPT* diplotypes (*p* = 0.895). After implementing Bonferroni to control for multiple comparisons, the relationship between the *APOE4* allele and cortical SUVRs remained significant (*p* = 0.020) (Table 3).

**Table 3:** Difference in mean cortical grey matter SUVRs based on genotype (mean ± standard deviation).

***Gene***	***N***	***Carrier SUVRs***	***Non-carrier SUVRs***	***Unadjusted p***	***Adjusted p***^a^
*APOE* (*APOE4* allele carriers/*APOE4* allele non-carriers)	13/25	1.38±0.10	1.27±0.14	0.010	0.020
*MAPT* (H1H1 diplotype/H1H2 or H2H2 diplotype)	21/17	1.31±0.13	1.31±0.14	0.895	N.S.

One-way ANCOVA, controlled for age; N.S. = not significant.

a Bonferroni adjusted p-value; significant at *p*<0.05.

### 3.5 The relationship between *APOE* and *MAPT* genotypes and neuropsychological assessments

The neuropsychological assessment results for the *APOE4* carrier/non-carrier groups are summarized in Table 1. The neuropsychological assessment results for the *MAPT* H2 carrier and non-carrier groups are summarized in Table 2. The independent student *t*-test showed no significant difference in the scores on TMT A & B, RAVLT, RVDLT, SDMT, digit span forward & backward, and PAI depression and aggression scores (all unadjusted *p*>0.06), between the *APOE4* carriers and non-carriers. No significant differences in the neuropsychological assessment scores (all unadjusted *p*>0.15) were found between *MAPT* H2 carrier and non-carrier groups.

### 3.6 Genotype counts between high and low cortical PET tau groups

**Fig. 1:**
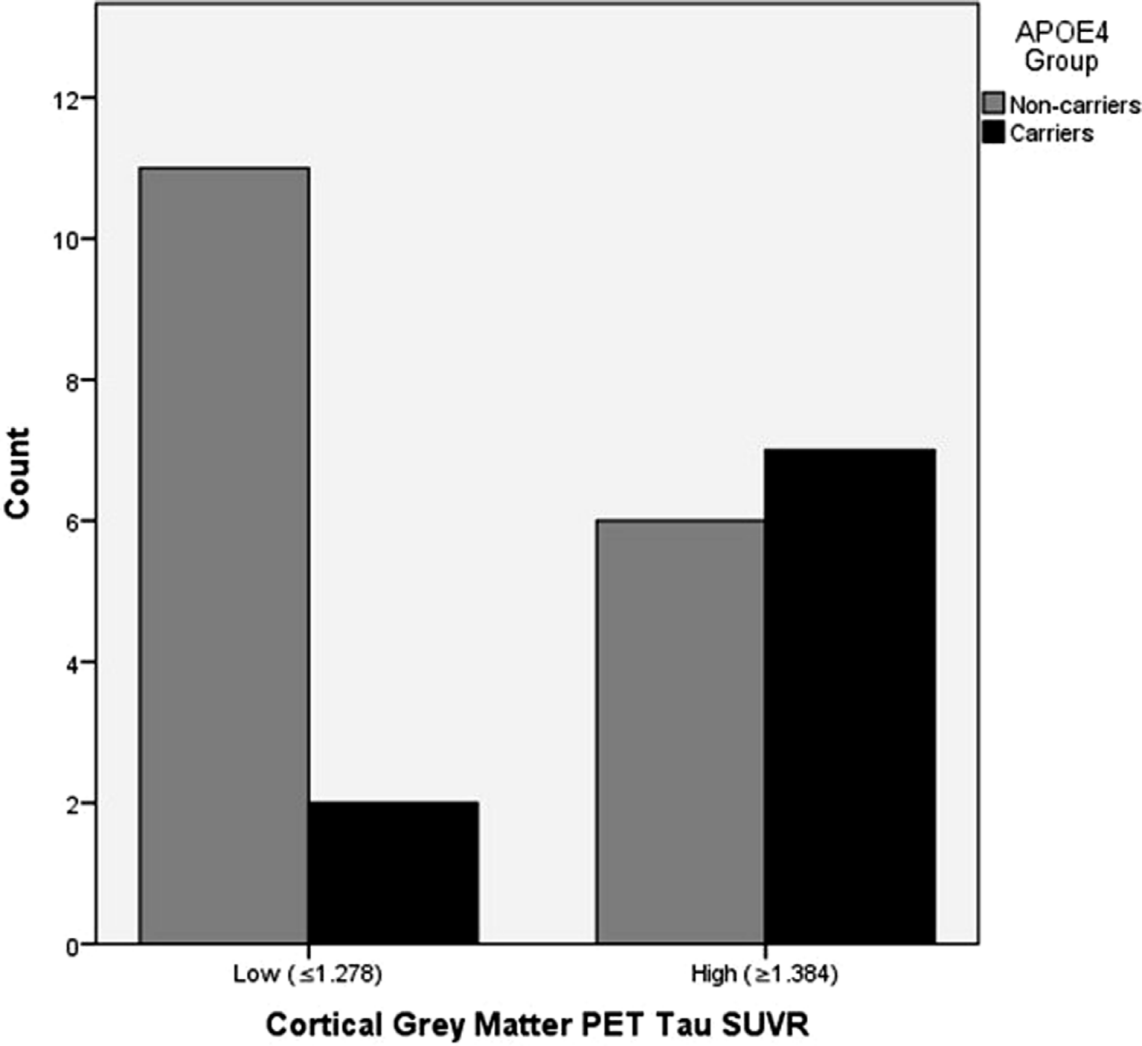
*APOE4* genotype counts amongst low (*N* = 13) and high (*N* = 13) cortical grey matter PET tau groups.

In order to compare the frequency of *APOE4* carriers and non-carriers according to cortical PET tau, we divided the entire cohort (*N* = 38) into three equal groups based on PET [F-18]AV-1451 SUVR values and dropped the middle group, leaving the low (*N* = 13; ≤1.278 SUVR) and high (*N* = 13; ≥1.384 SUVR) PET tau groups for comparison. The demographics of the high and low PET tau groups did not differ (Table 4). The independent student *t*-test showed no significant difference in the scores on TMT A & B, RAVLT, RVDLT, SDMT, digit span forward & backward, and PAI depression and aggression scores following Bonferroni correction, between the high and low cortical PET tau groups. Fisher's exact test showed a significantly higher frequency of *APOE4* allele carriers in the high cortical grey matter PET SUVR group (*p* = 0.048; one-sided) (Fig. 1). The demographics, neuropsychological assessment scores, and genotype counts for the middle tertile that was dropped from the statistical analysis is presented in Table 5.

**Table 4:** Demographics of the high and low cortical PET tau SUVR groups (mean ± standard deviation).

	***Low (≤1.278 SUVR)***	***High (≥1.384 SUVR)***	***Unadjusted p***	***Adjusted p***^c^
***Demographics***				
N	13	13		
Age (years)	57.00±14.23	49.92±14.19	0.22	N.S.
Concussion number	10.00±15.67	3.92±3.17	0.19	N.S.
	(min:0, max:60,	(min:0, max:10,		
	median:4)	median:4)		
No. of professional years played (years)	8.23±5.97	6.85±2.85	0.46	N.S.
	(min:2, max:21)	(min:0, max:10)		
No. of years since last reported concussion (years)	20.36±15.34^a^	20.21±17.23^b^	0.98	N.S.
	(min:3, max:54)	(min:0.5, max:61)		
PET Scanner	12 PET/CT; 1 HRRT	13 PET/CT	1.00	N.S.
***Neuropsychological Assessments***				
TMT A t-score	56.46±9.29	54.85±13.83	0.73	N.S.
	(0/13 impaired)	(1/13 impaired)		
TMT B t-score	54.62±12.69	57.23±11.10	0.45	N.S.
	(1/13 impaired)	(0/13 impaired)		
RAVLT Trials 1–5	0.08±0.87	−0.28±1.16	0.37	N.S.
z-score	(0/13 impaired)	(1/13 impaired)		
RVDLT Trials 1–5	−0.37±1.17	−0.45±1.05	0.85	N.S.
z-score	(3/13 impaired)	(3/13 impaired)		
SDMT Oral z-score	0.43±1.94	0.35±1.23	0.91	N.S.
	(0/13 impaired)	(1/13 impaired)		
SDMT Written	0.26±0.70	0.22±1.25	0.91	N.S.
z-score	(0/13 impaired)	(1/13 impaired)		
Digit Span	52.54±27.98	75.31±24.89	0.04	N.S.
Backwards%	(0/13 impaired)	(0/13 impaired)		
Digit Span Forward%	49.23±30.17	66.77±29.48	0.15	N.S.
	(1/13 impaired)	(0/13 impaired)		
PAI Depression	50.62±15.67	50.23±11.22	0.94	N.S.
t-score	(1/13 impaired)	(2/13 impaired)		
PAI Aggression	49.31±5.77	51.77±13.16	0.55	N.S.
t-score	(0/13 impaired)	(3/13 impaired)		

Independent student *t*-test, Fisher's exact test & Mann-Whitney U comparison; unadjusted significance level set at *p*<0.05 (2-sided). The number of participants with impaired scores is presented underneath the mean scores for each neuropsychological assessment in each group.

a Data is not included for 1 participant because he could not recollect the date of last concussion.

b Data is not included for 2 participants because they could not recall any concussions.

c Bonferroni adjusted p-value; significant at *p*<0.05.

**Table 5:** Demographics of middle tertile group based on cortical PET tau SUVR groups (mean ± standard deviation).

	***Middle Tertile (1.278 < and >1.384 SUVR)***
***Demographics***	
N	12
Age (years)	50.83±13.64
Concussion number	4.42±2.28
	(min:2, max:10,
	median:4)
No. of professional years played (years)	7.67±4.58
	(min:0, max:14)
No. of years since last reported concussion (years)	22.08±13.83
	(min:2, max:41)
PET Scanner	10 PET/CT; 2 HRRT
***Neuropsychological Assessments***	
TMT A t-score	51.58±8.40
	(0/12 impaired)
TMT B t-score	53.33±7.17
	(0/12 impaired)
RAVLT Trials 1–5	0.54±0.53^a^
z-score	(0/11 impaired)
RVDLT Trials 1–5	0.88±1.92^a^
z-score	(1/11 impaired)
SDMT Oral z-score	1.07±1.10^a^
	(0/11 impaired)
SDMT Written	0.43±1.04
z-score	(1/12 impaired)
Digit Span	79.64±20.58^a^
Backwards%	(0/11 impaired)
Digit Span Forward%	76.36±24.56^a^
	(0/11 impaired)
PAI Depression	51.00±13.92
t-score	(2/12 impaired)
PAI Aggression	54.92±13.02
t-score	(2/12 impaired)
**Genotype Counts**	
*APOE* (*APOE4* allele carriers/*APOE4* allele non-carriers)	4/8
*MAPT* (H1H1 diplotype/ H1H2 or H2H2 diplotype)	5/7

The number of participants with impaired scores is presented underneath the mean scores for each neuropsychological assessment.

a One participant's score is missing due to the refusal to undergo the full neuropsychological, testing, and a reduced battery was administered instead.

## 4 Discussion

To our knowledge, this is the first study to examine the relationship between *APOE, MAPT* and cortical tau burden as seen with PET [F-18]AV-1451 imaging in a cohort of former professional and semi-professional sport athletes with multiple concussions or sub-concussive hits at risk of delayed neurodegeneration, specifically CTE. The results of this study showed a significant association between the presence of an *APOE4* allele and higher cortical grey matter PET [F-18]AV-1451 SUVR, currently believed to be a marker of tau burden in AD. As well, *APOE4* carriers were more frequent amongst the high cortical PET tau group, compared to the low cortical tau group. No association was found between *MAPT* H1H1 carrier status and cortical grey matter PET [F-18]AV-1451 SUVR.

The exact direct or indirect mechanism that implicates *APOE* in tau burden is still unclear. *APOE* is present in the cytoplasm of nerve cells, where it may interact with other molecules in an isoform-dependant manner [Huang et al., 1995]. Tau is a microtubule-associated protein implicated in axonal transport [72], and previous findings show a decreased affinity of *APOE4* towards the microtubule-binding domain of tau protein [Huang et al., 1995]. This makes tau more vulnerable to being hyperphosphorylated, and therefore unable to bind microtubules, leading to its aggregation and consequently pathology [Terrell et al., 1]. Furthermore, *APOE4* showed increased binding to Aβ which is implicated in increased senile plaque formation in AD [Tierney et al., Nov, Strittmatter et al., 1]. Autopsy studies showed greater staining for senile plaques in the brains of *APOE4* homozygotes than *APOE3* homozygotes [Saunders et al., 1993, Strittmatter et al., 1]. In the most recent literature, tau and amyloid were proposed to work together synergistically to amplify each other's abnormal aggregation and subsequent tau-associated cognitive decline, specifically in the context of AD [Ittner and Götz, 2011 Feb] . In the current study, of the 9 participants who had CSF analysis all were negative for AD biomarkers. The remaining 29 participants showed no typical AD atrophy on MRI or tracer signal retention on PET, so there is no obvious evidence to suspect that the results of our study are due to an underlying AD pathology. Our cohort is that of former contact sport athletes at risk for neurodegeneration, especially CTE, and the pathophysiology behind CTE is mainly defined by abnormal aggregates of hyperphosphorylated tau. The exact pathophysiology behind the toxic function of tau aggregates remains unclear. However, it is hypothesized that abnormal aggregates of hyperphosphorylated tau disrupt the normal cellular transport within the axons, leading to synapse loss and ultimate neuronal death – resulting in disrupted neural circuits and eventual cognitive decline [Spillantini and Goedert, 2013 Jun 1]. Previous studies highlight a close relationship between tau pathology, neuronal loss and disease severity in AD and other tauopathies [Williams and Lees, 2009, Iqbal et al., 3]. The lack of association between tau burden and *MAPT* H1H1 may not be unexpected given that this diplotype prevalence is elevated in PSP and CBD, wherein the underlying tau pathology is a 4-repeat isoform tauopathy and a straight filament, whereas CTE is similar to AD with a mixture of both 3- and 4-repeat and a paired helical filament, and so very different [Woerman et al., 13].

The role of *APOE4* in concussion remains unclear. Most previous studies examining the potential effect of *APOE4* included TBIs of various severity in diverse populations, making between-study comparisons difficult. An association between *APOE4* alleles and concussion has been reported in college athletes [Terrell et al., 1, Tierney et al., Nov], and there is evidence for an increased risk of bleeding following TBI in *APOE4* carriers, which may prolong recovery [Tierney et al., Nov]. A prospective study in college athletes did not, however, find an association between *APOE4* and the risk of first concussion [Kristman et al., 2008]. amongst army veterans, *APOE4* allele carriers showed poorer performance on memory tasks following TBI compared to non-carriers but no difference in executive function [Crawford et al., 9]. A meta-analysis showed an association between *APOE4* and increased risk of poor outcome 6-months post TBI [Zhou et al., Apr]. However, another study using the same 6-month post TBI follow up duration found no relationship between *APOE4* and patient prognosis [Chamelian et al., 1]. Specific to athletes, the presence of *APOE4* has been associated with increased symptom reporting following a sport-related concussion [Merritt et al., 21] and boxers who were *APOE4* carriers showed worse neurological outcome [Jordan et al., 9]. There does not appear to be an increased risk of suffering a concussion in *APOE4* carriers [Terrell et al., 1, Tierney et al., Nov]. The results of our study are similar to previous research, where we found no significant association between *APOE4* and concussion history or performance on neuropsychological assessments, however, we did find that *APOE4* carriers had elevated tau burden as measured with PET [F-18]AV-1451.

There are a number of limitations to the current study. First, the small sample size and lack of a replication cohort limit the statistical power. As well, the total years of play for all athletes was not collected, missing an opportunity to examine the effect of total years of play on neuroimaging and fluid biomarkers. Next, participant cohort is highly varied with regards to age, concussion number, and performance on neuropsychological tests. There is also no matched healthy control group. Presence of a reliable control group with no history of contact sports would have provided a PET tau SUVR cut-off that could be used to divide participants into groups with normal and elevated tau burden. Furthermore, making a comparison between the high and low PET tau groups by dropping the middle third of the cohort decreased the total number of participants significantly, reducing the power for that specific analysis. Another limitation is the solely neuropathological nature of CTE diagnosis, leaving us unable to tell whether any of the participants have underlying CTE related changes. The results of this study are thus generalizable to former professional and semi-professional sport athletes at high risk of concussions with no evidence of active neurodegenerative changes. One limitation of the current study is lack of information with regards to race of included participants. Previous studies reported differences in *APOE* allele frequencies between populations [Eto et al., 1986, Seet et al., 1, Tang et al., 11, KB Rajan et al., 2019], and *APOE4* was found to be a determinant of AD risk in whites. Earlier studies reported that African Americans and Hispanics have an increased frequency of AD regardless of their *APOE* genotype, however the most recent literature showed that *APOE4* has a weak association with AD incidence amongst African Americans and Hispanics, in comparison to white populations [Tang et al., 11, Blue et al., 2019, Rajabli et al., 2018, Yu et al., 2017, KB Rajan et al., 2019]. With regards to *MAPT*, the H2 haplotype was reported to be almost exclusively Caucasian in origin [Evans et al., 21]. Finally, the use of cerebellar grey matter as a PET reference region has been widely studied and established for use in AD, but not in concussion. Cerebellar atrophy has been reported within a concussed cohort [Misquitta et al., 2018], and therefore the cerebellum might not be the ideal reference region in TBI cases. One study examining the [F-18]AV-1451 tracer in veterans with blast neurotrauma used a different reference region (ie. isthmus of cingulate) for its PET tau analysis [Robinson et al., 3] rather than the usual reference region of the cerebellum used in the athletes’ PET studies described above [Robinson et al., 3, Mitsis et al., 2014, Dickstein et al., Sep]. Further research is warranted in this area.

Overall, our results suggest a relationship between *APOE4* and tau burden as measured by [F-18]AV-1451 in the brain of athletes at risk for delayed neurodegeneration and CTE. A marked feature of CTE pathology is the abnormal aggregates of phosphorylated tau protein within the cortex in the form of NFTs. The increased tracer signal in the cortex of *APOE4* carriers could signify a neurodegenerative process and PET tau may be a biomarker for this process, but more research is needed to establish that.

## Authors’ roles

A.V. acquired the data, analysed the data, interpreted the data and drafted the manuscript for intellectual content. F.T. and C.B. analysed and interpreted the data. A.T., S.A.N, M.K., and R.G. had major roles in data acquisition. C.S. analysed and interpreted the data, revised manuscript for intellectual content. M.G. and D.M. analysed and interpreted the data. R.W. and D.M. interpreted the data and revised the manuscript for intellectual content. R.B. and B.C. acquired and interpreted the data, R.B. also revised the manuscript for intellectual content. K.D.D., P.R., S.H. and E.G. interpreted the data and revised the manuscript for intellectual content. C.T. had a major role in acquisition of data, interpreted the data and revised the manuscript for intellectual content. M.C.T. had a major role in acquisition of data, interpreted the data, drafted and revised the manuscript for intellectual content.

## Funding

Toronto General and Western Hospital Foundations; Weston Brain Institute; Canadian Consortium on Neurodegeneration in ageing; Krembil Research Institute. There was no role of the funders in this study.

## CRediT authorship contribution statement

**Anna Vasilevskaya:** Conceptualization, Methodology, Formal analysis, Writing - original draft. **Foad Taghdiri:** Methodology, Formal analysis. **Charles Burke:** Methodology, Formal analysis. **Apameh Tarazi:** Investigation. **Seyed Ali Naeimi:** Investigation. **Mozghan Khodadadi:** Investigation. **Ruma Goswami:** Investigation. **Christine Sato:** Formal analysis, Resources. **Mark Grinberg:** Formal analysis. **Danielle Moreno:** Formal analysis. **Richard Wennberg:** Formal analysis, Resources. **David Mikulis:** Formal analysis, Resources. **Robin Green:** Investigation, Formal analysis. **Brenda Colella:** Investigation, Formal analysis. **Karen D. Davis:** Writing - review & editing. **Pablo Rusjan:** Data curation, Writing - review & editing. **Sylvain Houle:** Writing - review & editing. **Charles Tator:** Investigation, Formal analysis, Writing - review & editing. **Ekaterina Rogaeva:** Formal analysis, Writing - review & editing. **Maria C. Tartaglia:** Investigation, Formal analysis, Writing - review & editing, Supervision.

## Declaration of Competing Interest

Authors report no conflicts of interest.
